# Arthroscopic Assisted Reduction and Percutaneous Fixation of Acute Perilunate Injuries

**DOI:** 10.1016/j.eats.2024.103350

**Published:** 2024-12-10

**Authors:** Rafi Husain, Jonathan Persitz, Andrea H.W. Chan, Ryan Paul

**Affiliations:** Hand Program, Division of Plastic, Reconstructive and Aesthetic Surgery, University Health Network, Toronto Western Hospital, affiliated with Temerty Faculty of Medicine, University of Toronto, Toronto, Ontario, Canada

## Abstract

Managing acute perilunate injuries (APLI) presents significant challenges due to the complex anatomy and technical intricacies involved. Despite open reduction and fixation being the gold standard treatment, the long-term prognosis remains guarded. Wide surgical exposures with extensive soft tissue releases may contribute to poorer functional outcomes, particularly with respect to range of motion. This surgical technique describes an approach employing arthroscopic-assisted reduction and fixation, suitable for both acute perilunate dislocation and fracture-dislocation injuries using minimally invasive methods.

Acute perilunate injuries (APLI), characterized by their complexity and severity, make up 7% of all carpal injuries.^1^ Regardless of the method of treatment, the prognosis for such injuries remains guarded because of the likelihood of significant stiffness, persistent pain, and post-traumatic arthritis.^2^ Historically, the gold standard for surgical management has been open reduction via dorsal and/or volar arthrotomies.^1^^,^^3^ This requires the open dissection of capsuloligamentous structures, which may disrupt critical secondary stabilizers of the carpus,^4^ increase the risk of stiffness/arthrofibrosis, and compromise blood supply.^5^^,^^6^, ^7^, ^8^ Arthroscopic assisted management offers an alternative that allows for minimally invasive direct assessment of the carpus.^2^^,^^5^

The described surgical technique outlines an arthroscopic assisted approach to the management of APLI, including perilunate dislocations and fracture dislocations.

## Surgical Technique

**Fig 1:**
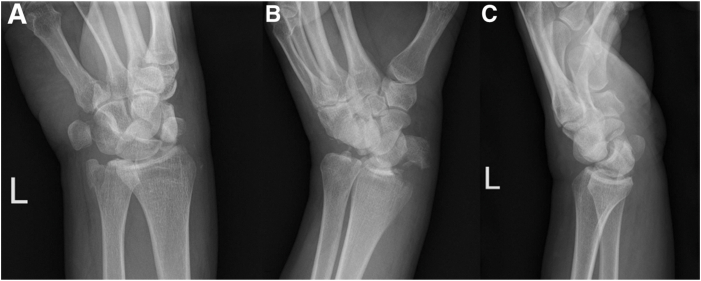
(A–C) Radiographic imaging of A 50-year-old left hand–dominant male, who sustained an injury to his left hand after a snowmobile accident. After assessment in the emergency department, x-ray imaging was ordered. PA, Oblique and lateral x-rays demonstrated a trans-radial, trans-ulnar styloid perilunate injury, with breaks in Gilula’s lines.

Initially, a complete workup of the patient is performed, including history, physical examination, and standard (PA, oblique and lateral) wrist injury radiographs (Fig 1). Computed tomography can also be performed to characterize carpal fractures and avulsions, evaluate for intra-articular loose bodies, and to assess for more subtle joint incongruities.

### Operating Room Setup

Our institutional preference for anesthesia is a regional brachial plexus block with sedation. For persistent dislocations, general anesthesia with paralysis may be employed to facilitate reduction.

**Fig 2:**
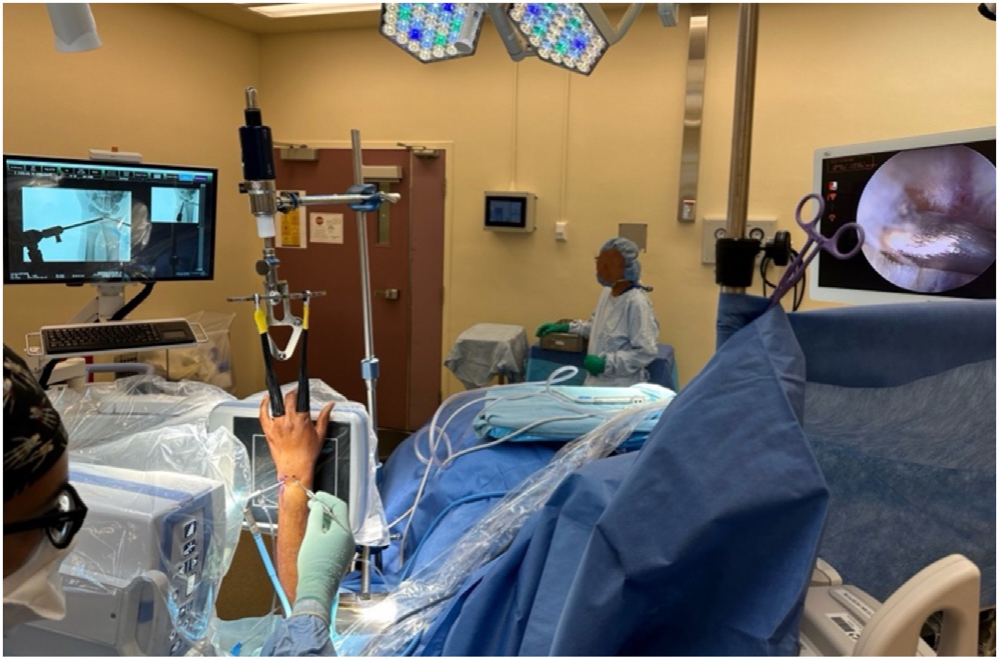
A demonstration of optimal room setup. The arm is abducted and the elbow flexed to 90° on a hand table. The hand is suspended in a traction tower (Smith & Nephew, London England) with finger traps. The arthroscopy monitor is positioned on the patient’s contralateral side, and a mini C-arm is positioned parallel to the hand table. The C-arm is withdrawn and advanced, as necessary, to facilitate concurrent arthroscopy of the wrist.

For arthroscopic visualization, the hand is suspended using an axial wrist traction tower and finger traps (Fig 2). The arm is abducted, and the elbow is flexed to 90°. Finger traps are applied to the index and ring fingers. At least 10 to 15 pounds of traction are applied. An Esmarch bandage is used to exsanguinate the limb, followed by inflation of a nonsterile tourniquet applied to the proximal arm. The base of the mini C-arm is positioned parallel to the hand table. When the hand is suspended in the traction tower, the mini C-arm is horizontally oriented, with the image intensifier parallel and as close as possible to the wrist. The mini C-arm can be advanced or withdrawn as needed during the procedure.

### Step 1: Provisional Reduction of Fractures

When possible, associated carpal fractures are managed with percutaneous headless compression screws. Alternative fixation options are considered on a case-by-case basis.

**Fig 3:**
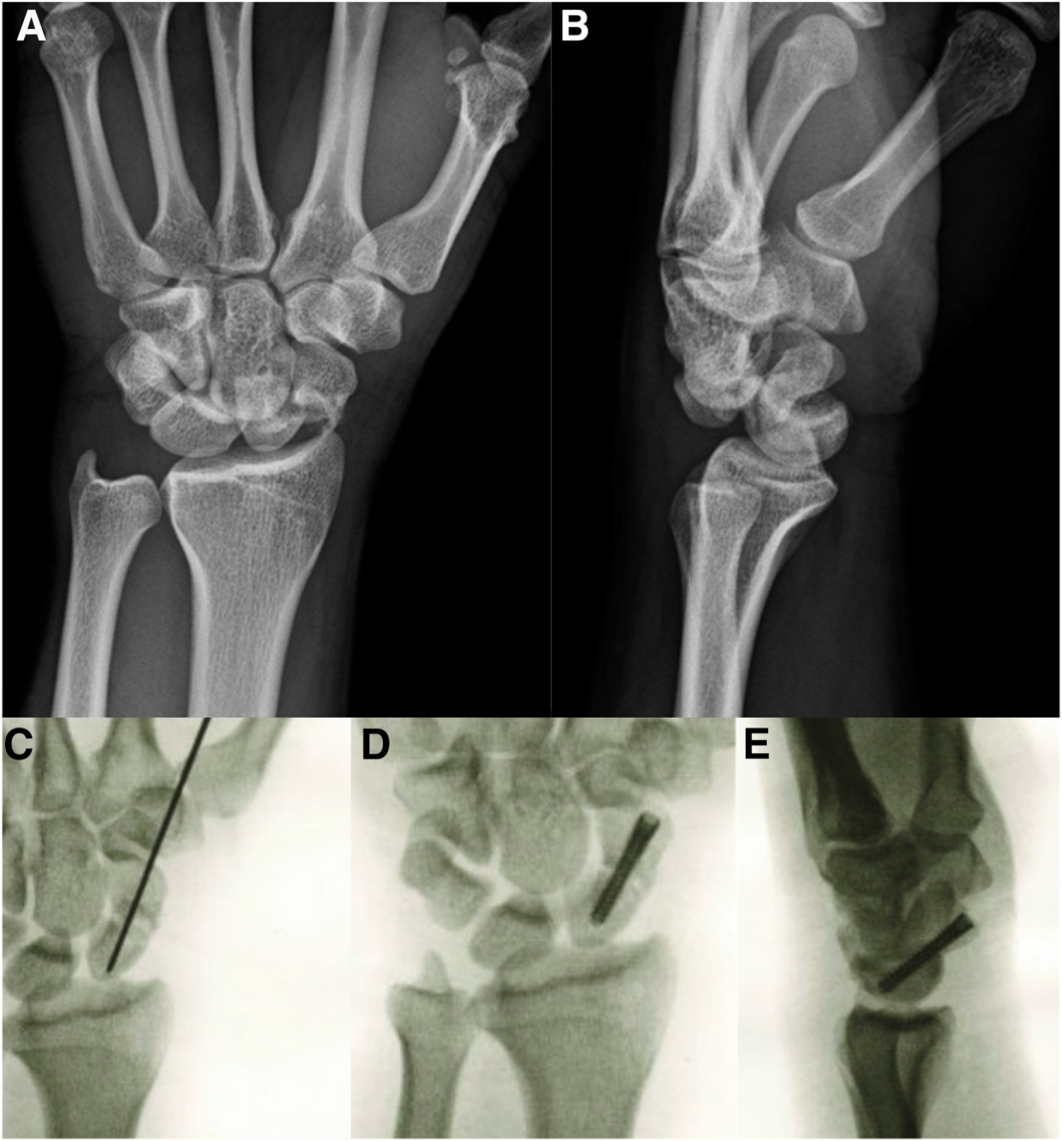
Prereduction radiographs of a left transscaphoid perilunate injury (A and B) sustained by a 24-year-old male while playing basketball. After closed reduction is performed, the patient is taken to the operating room for arthroscopic assisted management of the APLI. The scaphoid fracture is addressed initially. This was performed via a percutaneous volar technique. Intraoperative fluoroscopy demonstrates reduction and stabilization of the scaphoid with a Kirschner wire (C), followed by the insertion of a headless compression screw, with anatomic reduction of the scaphoid (D and E).

For scaphoid waist fractures, the senior author’s preference for percutaneous fixation is a retrograde volar-to-dorsal trajectory (Fig 3). Prior to suspending the hand with the traction tower, a preliminary reduction maneuver of intercarpal supination, wrist extension, and ulnar deviation is performed to reduce the scaphoid. Initial central wire placement is started in the distal pole and advanced to the fracture site without crossing it. While closed reduction might appear satisfactory under fluoroscopy, achieving anatomical reduction through this maneuver alone is uncommon. Therefore, we routinely proceed with traction and diagnostic arthroscopy (see step 2) to assess the fracture site prior to definitive fixation. During this process, we meticulously decompress the fracture sites and remove any debris to enhance the likelihood of achieving anatomical alignment. Once this is completed, improved ability to manipulate the fracture into a reduced position is possible and provisional fixation can be obtained by advancing the previously placed central guidewire. Reduction is confirmed both arthroscopically and fluoroscopically, and insertion of headless compression screw is performed.

**Fig 4:**
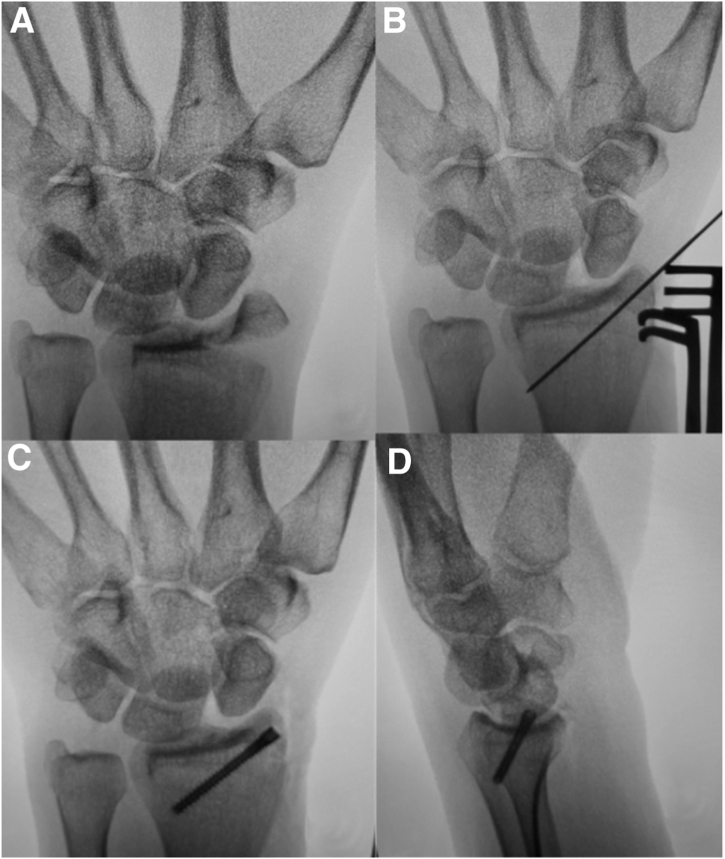
(A) Intraoperative fluoroscopy a trans-radial, trans-ulnar styloid perilunate injury after closed reduction maneuver, demonstrating improved carpal alignment. (B) Subsequent reduction of radial styloid fracture after a mini-open approach, and provisional fixation with a K-wire, performed prior to arthroscopy. (C and D) PA and lateral films demonstrating definitive fixation of the radial styloid fracture with headless compression screw. Now, the greater arc injuries have been addressed, attention will be turned to reducing the SL interval and the DISI deformity arthroscopically.

Fixation of other associated fractures can be addressed in a similar manner with percutaneous or mini-open incisions (Fig 4).

### Step 2: Diagnostic Arthroscopy and Adhesiolysis

**Fig 5:**
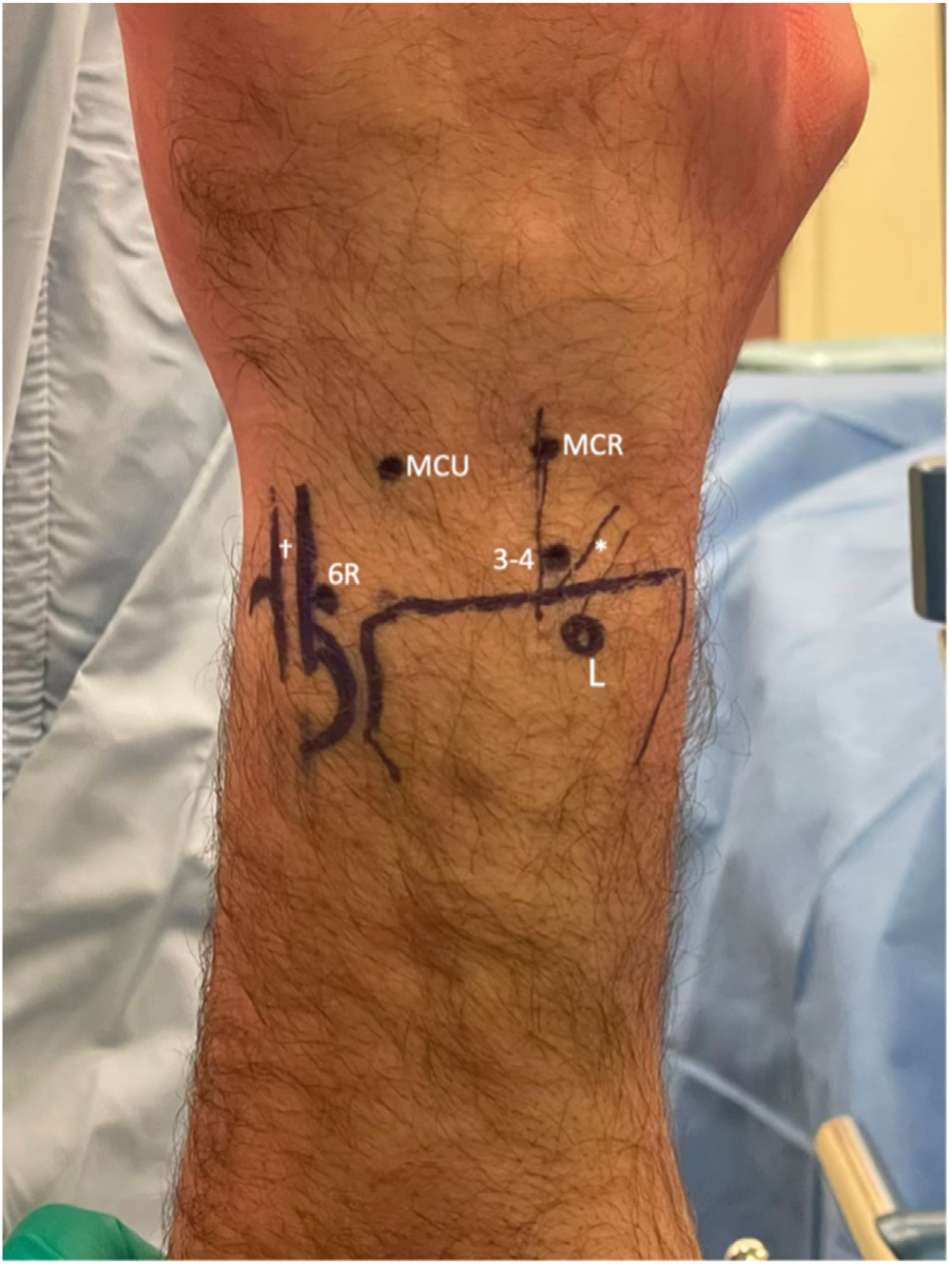
Surface markings for arthroscopy portals. The outline of the distal radius and ulna is drawn out, as well as the tendons of extensor pollicis longus (EPL) and extensor carpi ulnaris (ECU). Our primary radiocarpal viewing portal, the 3-4, is identified distal to Lister’s tubercle (L) and ulnar to the EPL tendon (∗). We prefer to use the 6R portal for secondary radiocarpal access. It is located just radial to ECU tendon (†). The midcarpal portals are identified roughly 1 cm distal to the 3–4 portal, and on the radial and ulnar sides of the capitellar neck (MCR and MCU, respectively).

**Fig 6:**
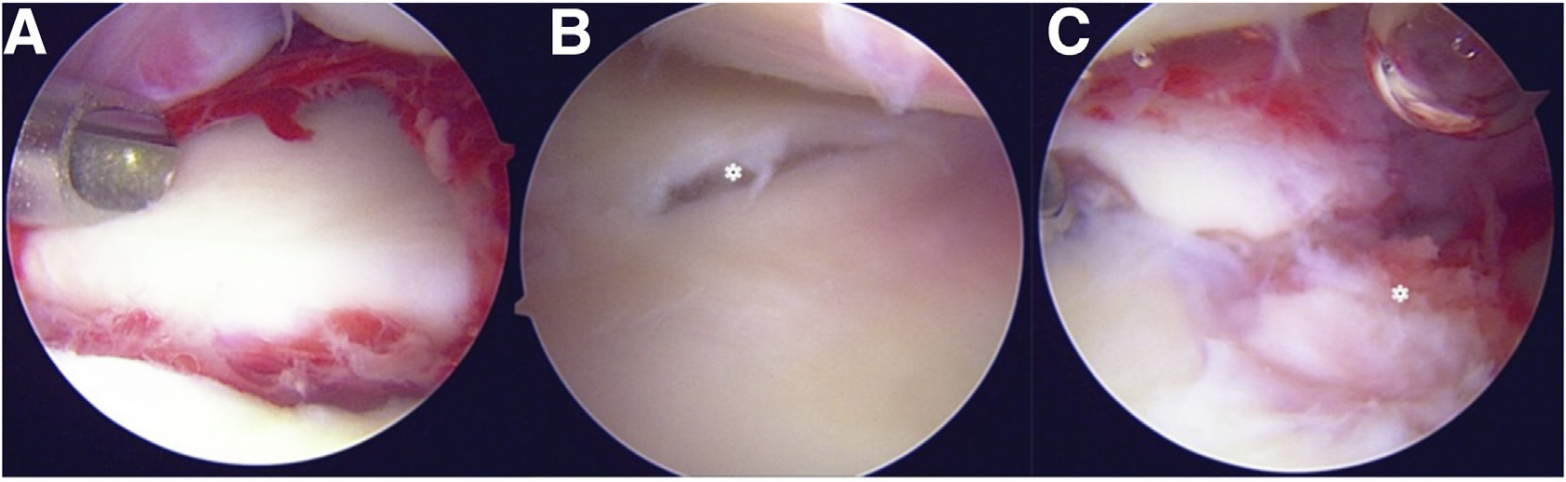
Diagnostic arthroscopy of the radiocarpal joint in our patient with a trans-radial, trans-ulnar styloid perilunate injury using the 3-4 portal for visualization. (A) Radial styloid fracture reduction after initial fixation via a mini-open approach to the radial styloid. (B) Diagnostic arthroscopy also demonstrated a central TFCC tear (∗), associated with the ulnar styloid fracture. (C) A volar rim fracture via arthroscopy (∗) of the distal radius was also identified, which was not fixed.

Standard wrist arthroscopy portals, including the 3-4, 6-R, mid-carpal radial (MCR) and mid-carpal ulnar (MCU) portals are marked (Fig 5). Initially, the joint is insufflated with a needle and syringe. Hematoma is flushed out with serial injection and aspiration until fluid is clear. This step is crucial for hematoma decompression, as well as to aid in visualization. The 3-4 portal is then established and a short 2.7-mm 30° arthroscope attached to a hand-pump small joint purging system is introduced. The 6-R portal is then established under direct visualization. A standard diagnostic arthroscopy is performed (Fig 6), including assessment of the radiocarpal articular surfaces and ligamentous structures. We evaluate the scapholunate and lunotriquetral ligaments, as well as the volar and dorsal extrinsic ligaments (i.e., radioscaphocapitate, long/short radiolunate, dorsal intercarpal ligament (DIC), and dorsal capsulo-scapholunate septum), which may be intact, associated with small avulsion fractures, or have insertional/midsubstance ruptures with displacement into the joint. A shaver is used to debride any synovium, torn ligaments, and bony/chondral debris.

**Fig 7:**
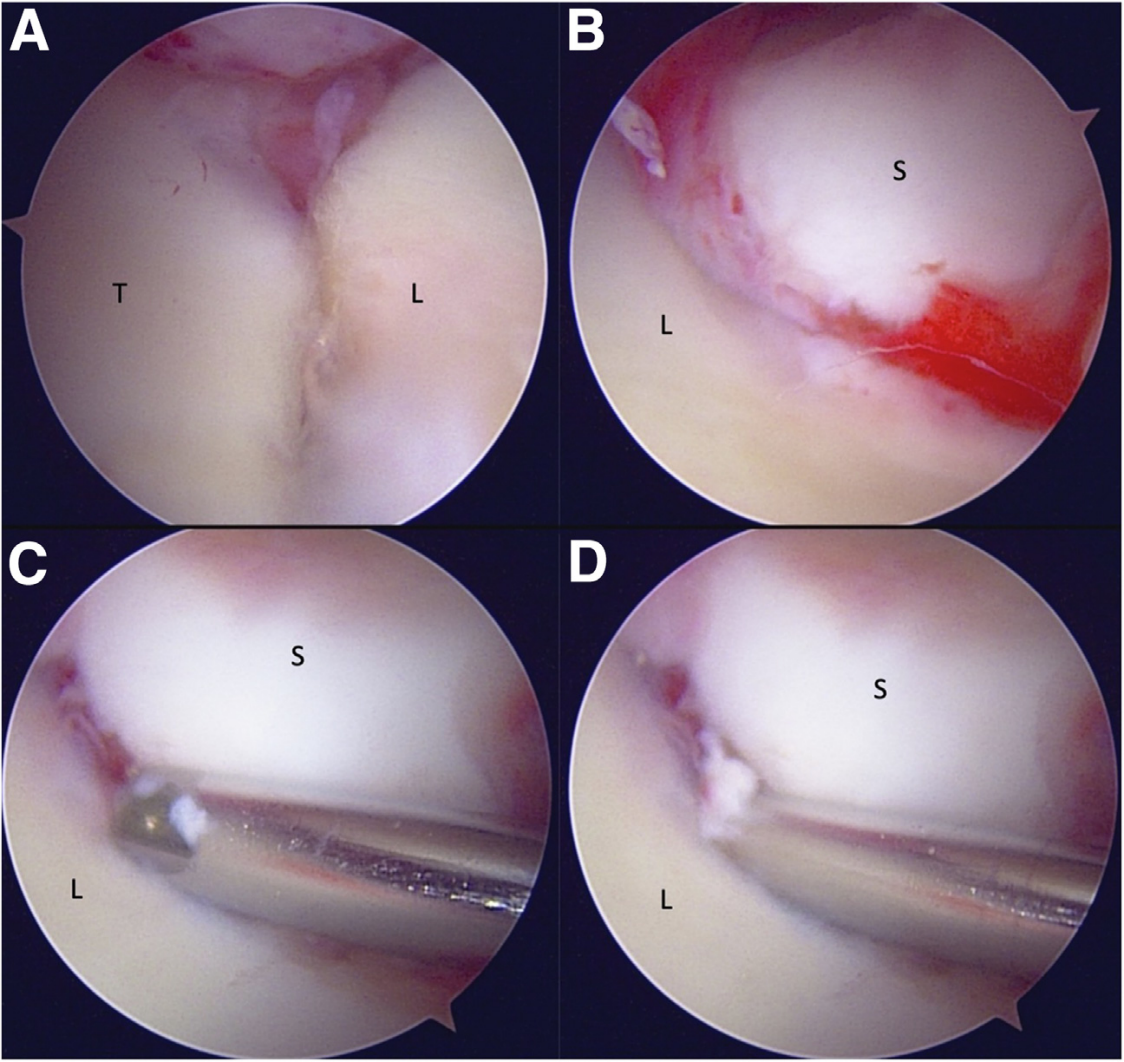
After diagnostic arthroscopy of the radiocarpal joint, we move to assessment of the midcarpal articulations. Visualization is obtained via the MCR portal. (A and B) Lunotriquetral and scapholunate intervals. Note the malalignment with step-off at the SL interval in B. (C and D) Incompetence of the SL ligament, as a 2-mm shaver as well as the arthroscope may be easily passed through the interval, indicating a Geissler stage IV injury.

The midcarpal portals are subsequently established. Through the midcarpal portals, the carpal bones are assessed for fracture/chondral damage, as well as the distal insertions of RSC and ulnocapitate ligaments volarly (part of the arcuate ligament complex). Dorsally, the deep attachments of the DIC can be assessed at the margins of the scaphoid, lunate, and triquetrum. Avulsions or midsubstance ruptures may be noted and synovium, debris, and torn ligament structures are debrided. The scapholunate (SL) and lunotriquetral (LT) intervals are then visualized and Geissler’s grading system is used to evaluate the degree of instability^9^ (Fig 7).

### Step 3: Scapholunate Reduction and Stabilization

**Fig 8:**
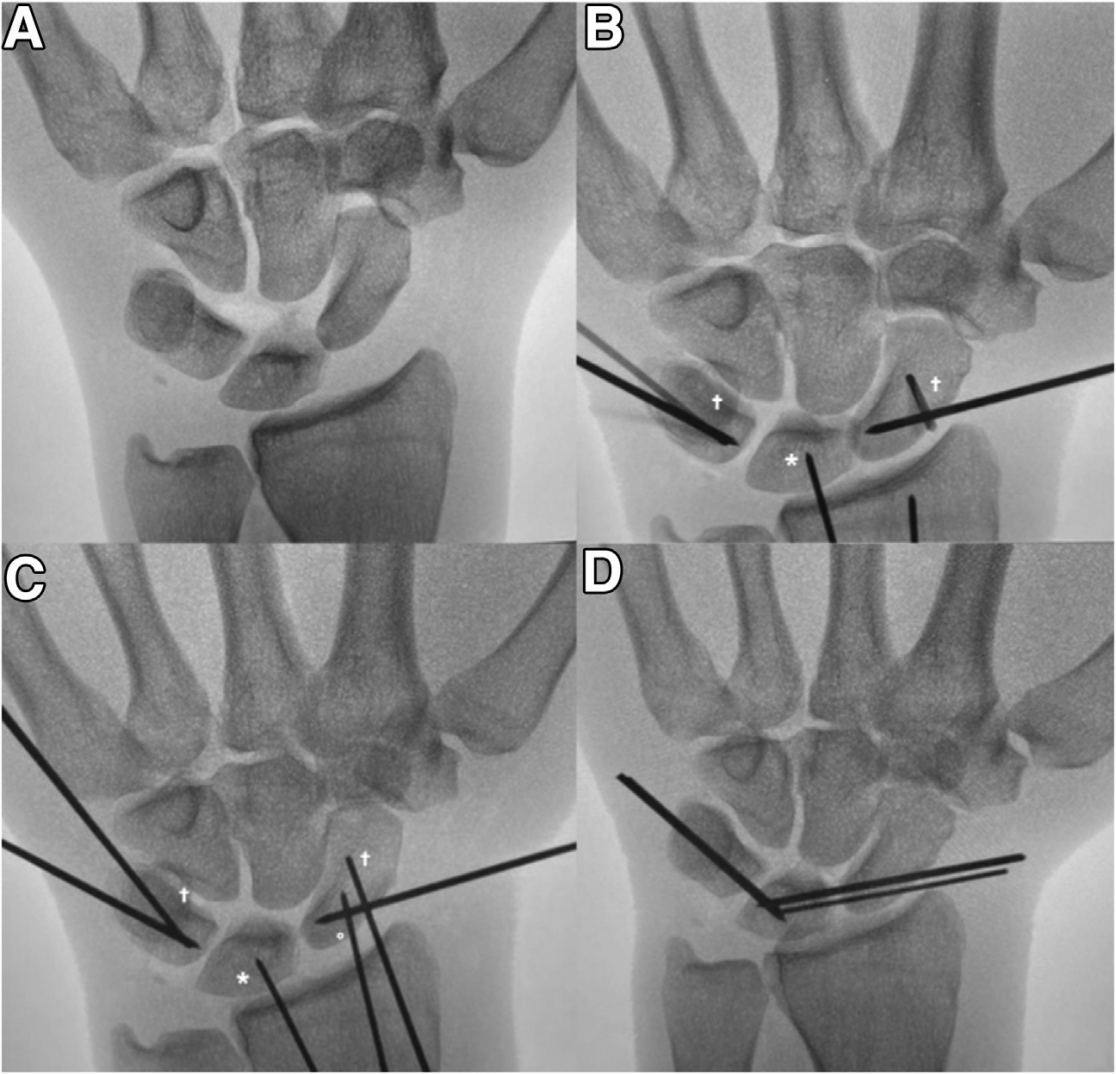
A 44-year-old male presenting with an acute a lesser arc perilunate injury (A) with incongruity of the SL and LT articulations. (B) As per the Linscheid maneuver, the K-wire is inserted across the radiolunate articulation (∗) after correction of the DISI deformity to stabilize the lunate and create a stable base for reduction of the carpus using joysticks (†). (C) A second radiocarpal wire across the radioscaphoid articulation is placed, to maintain reduction (°). K-wires are advanced through the scaphoid and triquetrum in preparation for definitive fixation. The carpus is then reduced under arthroscopic visualization, and these wires are advanced into the lunate with the carpus in a reduced position. (D) The radiolunate pins and joysticks are removed, with the remaining K-wires cut and buried subcutaneously.

Continuing with the MCU portal for visualization, we proceed with reduction of the scapholunate (SL) joint. Correction of lunate malposition (commonly flexion) may be achieved using the Linscheid maneuver, wherein a 0.062-inch K-wire is advanced from the dorsal radius into the lunate, while being held in a neutral position under fluoroscopic control.^10^ This step is particularly useful in APLIs, where there are combined intrinsic/extrinsic ligament injuries resulting in near-circumferential soft tissue disruption about the lunate. The maneuver creates a stable base for reduction and fixation of the remainder of the carpus. After this, multiple additional 0.045-inch K-wires are advanced into the scaphoid, lunate, and/or triquetrum and used as “joysticks” for obtaining reduction as needed (Fig 8). Fixation is then performed, beginning with the SL interval. Using fluoroscopy, two parallel provisional K-wires are advanced across the scaphoid aiming toward the lunate articulation. A combination of manual and joystick manipulation is then performed to reduce the SL interval—correcting the typical flexed, pronated, and dorsally translated deformity of the scaphoid. A trocar or blunt elevator can be used via the MCR portal to help fine tune the reduction under arthroscopic guidance, and then K-wires are advanced across the interval and into the lunate. If there is any concern for persistent scaphoid instability into flexion, an additional K-wire is percutaneously placed between the scaphoid and capitate; however, typically, this has not been necessary.

### Step 4: Lunotriquetral Reduction and Stabilization

Attention is then turned to the LT articulation using the MCR portal for visualization. The most common pattern of displacement noted by the senior author is dorsal translation and coronal malrotation (loss of ulnar inclination). Using fluoroscopy, the surgeon advances 2 parallel K-wires across the triquetrum starting ulnarly and just below the articular surface. Reduction is obtained using manual reduction (dorsal to volar digital pressure) and manipulation with joysticks (to restore ulnar inclination). Close attention is paid to lunate morphology to ensure reduction is aligned to the hamate facet of the lunate when a type 2 lunate is present. Once reduction is confirmed arthroscopically, K-wires are advanced across the triquetrum into the lunate, from an ulnar to radial direction. Once satisfied with the final reduction and fixation arthroscopically and fluoroscopically, K-wires are then buried subcutaneously. Traction is released, and we additionally employ live fluoroscopy and gentle passive range of motion to ensure stability.

### Step 5: Postoperative Care

**Fig 9:**
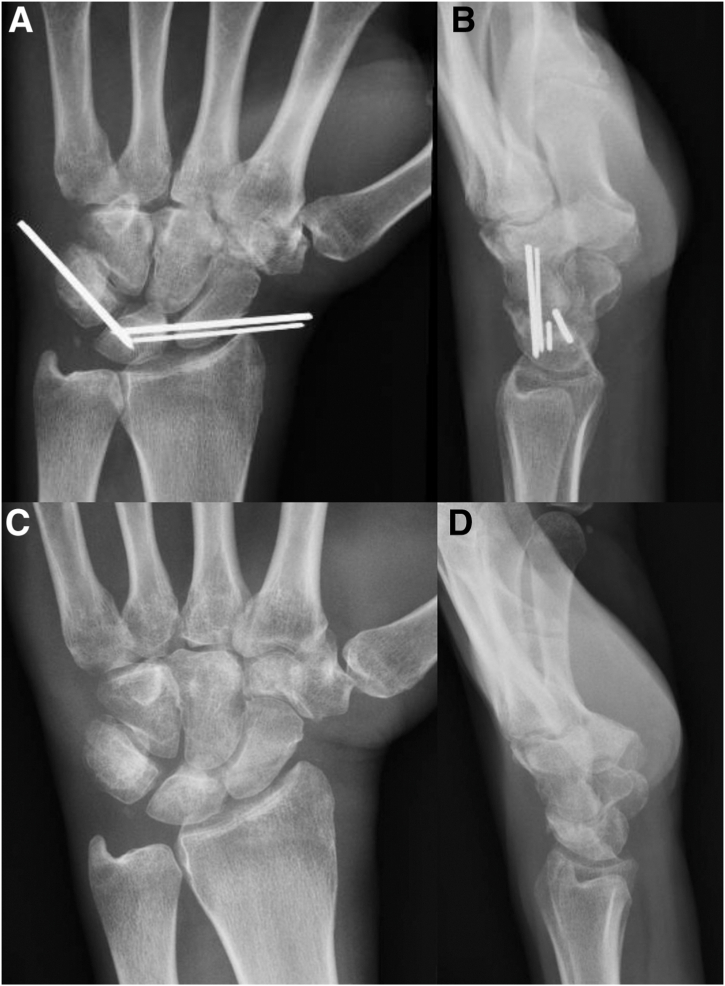
PA and lateral radiographs of our patient with an acute lesser arc perilunate injury 2 weeks postoperatively in fiberglass cast (A and B), with subsequent removal of buried K-wires in clinic 8 weeks postoperatively. (C and D) PA and lateral x-rays of patient demonstrating restoration of Gilula’s arcs and maintained SL and LT intervals, 3 months postoperatively.

Postoperatively, the patient is placed into a below-elbow volar resting splint, which is transitioned to a circumferential fiberglass cast for a total of 6 weeks, followed by a removable wrist splint. K-wires remain in situ until approximately 6–8 weeks, at which point they are removed under local anesthesia in the clinic (Fig 9). Progressive range of motion and gradual strengthening are then undertaken (Video 1).

## Discussion

Arthroscopic management of APLI was first described in 1995,^11^ and although its use is gaining popularity, previous reports have been limited to a small number of case series without detailed elaboration on surgical steps.^5^^,^^6^^,^^8^ Our article further describes the techniques used, with accompanying insights from a senior author experienced in arthroscopy.

Long-term outcomes of arthroscopically assisted management of APLI remain unclear. A systematic review by Liechti et al. highlighted a decreased complication rate compared to open approaches.^2^ Furthermore, there is evidence to suggest that arthroscopically managed APLIs have a superior range of motion compared to open techniques. These studies, however, are small and retrospective in nature.^12^

Pearls and pitfalls for this technique are included below (Table 1).

**Table 1:** Key Technical Pearls for the Arthroscopically Assisted Management of APLI

Steps	Technical Pearls
Assess and treat fractures	• Percutaneous or mini-open approach addresses greater arc injuries. • Utilize arthroscopy to confirm reduction and hardware placement. • Use headless compression screws when feasible. • Stabilization of bony injuries greatly increases stability prior to addressing ligamentous component of APLI.
Diagnostic arthroscopy and adhesiolysis	• Flushing joint hemarthrosis is crucial for visualization. • Perform thorough diagnostic arthroscopy to identify associated injuries (i.e., TFCC, chondral lesions). • Assess the degree of instability of SL and LT intervals using probe. • Debride any redundant capsule/torn ligament or chondral debris.
SL/LT reduction and stabilization	• Consider radiocarpal pins (i.e., Linscheid pin) to correct DISI deformity and to temporarily stabilize lunate/scaphoid (± scaphocapitate). • Joysticks can be used to manipulate and reduce carpus. • Strategic manual reduction maneuvers are essential. • Arthroscopic probe or blunt trocar can be used to further fine-tune reduction. • “Preload” K-wires into scaphoid and triquetrum and advance them once satisfied with reduction under direct arthroscopic visualization • Wires can be buried subcutaneously

NOTE. As with other arthroscopic techniques, arthroscopic management of perilunate injuries involves a significant learning curve.

Although open arthrotomy presently remains the gold standard for APLIs, arthroscopic management offers several advantages, including a minimally invasive approach that could prove to be an equally or even more effective method of fixation.

## Disclosures

The authors (R.H., J.P, A.C., R.P.) declare that they have no known competing financial interests or personal relationships that could have appeared to influence the work reported in this article.

## Supplementary Data

Video 1 A case demonstration of our arthroscopic technique for the management of acute perilunate injuries (APLI). A 44-year-old male sustained an acute ligamentous APLI, that was reduced and stabilized with arthroscopic assistance. We outline our preferred operating room setup, as well as our approach to maximize visualization and technical pearls to aid in reduction and fixation.
